# Early hypothermia improves survival and reduces the rise of serum biomarkers after traumatic brain injury in swine

**DOI:** 10.1186/cc12257

**Published:** 2013-03-19

**Authors:** M Kumar, AD Goldberg, M Kashiouris, L Keenan, A Rabinstein

**Affiliations:** 1 Mayo Clinic, Rochester, MN, USA

## Introduction

Poor outcomes in clinical trials on the use of therapeutic hypothermia following traumatic brain injury (TBI) may be due to the delay in reaching target temperature [1]. We hypothesize that early and rapid induction of hypothermia will mitigate neuronal injury and improve survival in a swine model of TBI.

## Methods

Twenty domestic cross-bred pigs (34 to 35 kg) were subjected to a 5 ATM (100 ms) lateral fluid percussion TBI. The brain temperature and ICP were measured using Camino^®^. Serum biomarkers for neuronal injury - S-100β, neuron-specific enolase, glial fibrillary acid protein (GFAP), and neurofilaments heavy chain - were measured daily using enzyme-linked immunosorbent assay. Twelve of the injured animals were rapidly cooled to 32°C within 90 minutes of the injury using a transpulmonary hypothermia technique [2]. Hypothermia was maintained for 48 hours. Eight injured control animals were maintained at 37°C. In both groups, anesthesia (isoflurane 1%) was discontinued and the animals were weaned off the ventilator after 48 hours. Five days post injury, the surviving animals were euthanized and necropsied. The data were analyzed using a log-rank (Mantel-Cox) test, and ANOVA.

## Results

Ten of the 12 hypothermia and four of the eight normothermia animals survived to the end of the 5-day study (χ^2 ^= 2.597, df = 1, *P = *0.1071). Although the probability of type I error between survival curves was 11%, the study was clinically significant and showed a clear trend toward improved survival with hypothermia. The intracranial pressures were significantly (*P <*0.05) lower in the hypothermia group. Both interventions - that is, general anesthesia and hypothermia - mitigated the rise of serum biomarkers following TBI. However, the suppression of biomarkers was sustained during the recovery period only in the hypothermia group. With the exception of the GFAP levels, the curves of all biomarkers were significantly different between the groups.

## Conclusion

Our preliminary findings show early initiation, rapid induction, and prolonged maintenance (48 hours) of cerebral hypothermia to lower intracranial pressure, blunt the rise in serum biomarkers, and improve survival following TBI.
